# Effect of Streptozotocin on Plasma Insulin Levels of Rats and Mice: A Meta-analysis Study

**DOI:** 10.3889/oamjms.2015.093

**Published:** 2015-08-25

**Authors:** Burcu Koksal

**Affiliations:** Inonu University, Department of Physiology, Inonu University, Faculty of Medicine, Malatya 44280, Turkey

**Keywords:** Streprozotocine (STZ), diabetes mellitus, serum insulin levels, meta-analysis

## Abstract

**BACKGROUND:**

In the studies focusing on diabetic organisms, Streprozotocine (STZ) is a frequently used agent to induce diabetes in rats and mice. However the current studies do not represent practical importance of their statistical findings. For showing practical importance of the differences in plasma insulin levels of diabetic rats and mice induced by STZ, there should be a statistical synthesis regarding statistical findings of the studies.

**AIM:**

The purpose of this study is to make a meta-analysis of the studies on the effect of STZ on plasma insulin levels in diabetic rats and mice.

**MATERIALS AND METHODS:**

In this study 39 effect sizes (37 studies) about levels of plasma insulin were analyzed by calculating individual effect sizes (d) and mean effect size.

**RESULTS:**

The effect sizes were between -13.7 and +65.3 and the mean effect size value (+9.33) represented a large effect indicating that STZ was an effective agent to significantly decrease plasma insulin levels of diabetic rats and mice.

**CONCLUSION:**

It can be said that the differences in plasma insulin levels between STZ-applied and no application groups has a practical importance in making animal model of diabetes.

## Introduction

Nowadays, diabetes is frequently seen in society, its prevalence is about 382 million people around the world [1]. Diabetes is characterized by insufficient secretion rate of insulin or lack of insulin activity [2, 3]. Diabetes is associated with different health problems including cardiovascular diseases, neuropathy, retinopathy, ulcers and amputations [4, 5]. Treatment of diabetes is a complex issue but some animal models were developed to understand the management as diabetes is a chronic condition [6, 7]. Over 30 years, alloxan, streptozotocin (STZ, 2-deoxy-2-(3-(methyl-3- nitrosoureido)-D-glucopyranose), high-fat diet-fed and nicotinamid are used for establishing experimental diabetes models of animal [8].

Streptozotocin is still commonly used agent to induce diabetes in rats and mice [9-12]. STZ is produced by Streptomycetes sachromogenes and STZ causes to abnormal B-cell functions by imparing glucose oxidatation and decreasing insulin biosynthesis and secretion [13, 14]. Szkudelski stated that STZ dose range is larger than alloxan and other agents and only one dose is enough to induce diabetes [15]. Decrease in plasma insulin levels in animal models after STZ application is used a sign for inducement of diabetes [16-18]. In spite of reporting significant differences in plasma insulin levels after STZ application, majority of the studies using STZ do not report practical importance or effect sizes of the differences. But there is a need to show practical importance for future decisions on dose and time of STZ application.

Based on this idea, the purpose of this study is to make a meta-analysis of the studies on the effect of STZ on plasma insulin levels in diabetic organisms.

## Materials and Methods

In this study, meta-analysis approach was used to evaluate practical importance of the differences regarding plasma insulin levels of STZ-induced diabetic rats and mice. Meta-analysis is different from a review including summarizing existent literature, since meta-analysis involves statistically synthesizing results of different studies [19, 20]. For meta-analysis in this study, Cohen’s d effect size values were calculated for 37 studies and mean effect size value was found for deciding about average effect size value as an indicator of mean practical importance of the differences in plasma insulin levels induced by STZ.

### Selection of the Publications

In selection process of the publications PubMed, Google Scholar, Proquest and National Theses Database System were searched by using key words “Plasma insulin levels, STZ, Rats”. The time restriction for the publications was 2005-2015. In National Theses Database System no thesis was found about the keywords it might be related to system error while Proquest search showed 89 theses. However, one thesis was found appropriate. When Pubmed was searched 526 results were found. As the highest publication number, Google scholar search results gave 3960 publications.

After adding the publications to the pool, checking abstracts and content of the publications were conducted. Eventually it was determined that 37 studies reported change in plasma insulin levels of diabetic organisms and they reported 39 differences for effect size calculations across different doses of STZ. Descriptive knowledge about the publications is represented in Table 1. The titles of them can be seen in appendix (Table 3).

**Table 1:** Descriptions of the publications in this study

Publication Date	Name of Journal or Institution	Subject	STZ Amount in the Application	Time between STZ application and plasma insulin measurement
2005	Biochemical and Biophysical Research Communications	Rats	65 mg/kg	9 days
2005	Pharmacological Research	Rats	50 mg/kg	4 weeks
2005	Journal of Ethnopharmacology	Rats	65 mg/kg	15 days
2005	Journal of Biochemistry and Molecular Biology	Rats	50 mg/kg	6 weeks
2006	Clinical and Experimental Pharmacology and Physiology	Rats	55 mg/kg	21 days
2006	Journal of Health Sciences	Rats	55 mg/kg	30 days
2006	Molecular and Cellular Biochemistry	Rats	100 mg/kg	45 days
2006	Basic & Clinical Pharmacology & Toxicology	Rats	50 mg/kg	45 days
2006	Clinical and Experimental Pharmacology and Physiology	Rats	55 mg/kg	30 days
2006	Diabetes	Mice	90-100 mg/kg	3 weeks
2006	Phytotherapy Research	Rats	50 mg/kg	21 weeks
2007	International Journal of Biological Macromolecules	Rats	50 mg/kg	30 days
2007	Journal of Ethnopharmacology	Rats	55 mg/kg	21 days
2008	Experimental Diabetes Research	Rats	45 mg/kg	8 weeks
2008	BMC Molecular Biology	Rats	65 mg/kg	15 days
2008	Atherosclerosis	Rats	60 mg/kg	7 weeks
2009	Clinical and Experimental Ophthalmology	Rats	60 mg/kg	1 week
2010	Phytomedicine	Rats	60 mg/kg	6 weeks
2010	Pharmacognosy Res.	Rats	55 mg/kg	15 days
2010	Archives of Medical Research	Rats	60 mg/kg	32 weeks
2011	Chemico-Biological Interactions	Rats	50 mg/kg	7 days
2012	International Journal of Endocrinology	Rats	65 mg/kg	5 days
2012	The Journal of Pharmacology And Experımental Therapeutıcs	Rats	60 mg/kg	24 weeks
2012	*West Virginia University, School of Medicine*	Mice	50 mg/kg	5 weeks
2012	Turkish Journal of Medical Sciences	Rats	45 mg/kg	8 weeks
2013	BMC Complementary and Alternative Medicine	Rats	55 mg/kg	3 days
2013	Diabetology & Metabolic Syndrome	Rats	50 mg/kg	60 days
2014	BMC Pharmacology and Toxicology	Rats	50 mg/kg	1 week
2014	Acta Histochemica	Rats	40 mg/kg	72 hours
2014	European Journal of Pharmacology	Rats	50 mg/kg	1 week
2014	Phytomedicine	Rats	40 mg/kg	4 weeks
2014	Pain Medicine	Rats	30 mg/kg	2 weeks
2014	Pain Medicine	Rats	35 mg/kg	2 weeks
2014	Pain Medicine	Rats	40 mg/kg	2 weeks
2014	Food and Chemical Toxicology	Rats	40 mg/kg	28 days
2015	International Journal of Experimental Pathology	Rats	45 mg/kg	24 hours
2015	Pharmacognosy Research	Rats	90 mg/kg	10 weeks
2015	Nutrition	Rats	35 mg/kg	72 hours
2015	Renal Failure	Rats	60 mg/kg	5 weeks

**Table 2:** Descriptive Values regarding Plasma Insulin Levels, Unit of Plasma Insulin Levels and Individual Effect Sizes of the Differences in the Publications

Publication Date	Name of Journal or Institution	Plasma Insulin Level in Control Group	Plasma Insulin Level in STZ-induced Diabetic Group	Unit of Plasma Insulin Level	Effect Size
2005	Biochemical and Biophysical Research Communications	3.11 ± 0.67	0.34 ± 0.11	ng/ml	5.8
2005	Pharmacological Research	57 ± 5	58 ± 4	mU/L	0.2 (-)
2005	Journal of Ethnopharmacology	35.40 ± 2.17	6.75 ± 0.15	μU/mL	18.7
2005	Journal of Biochemistry and Molecular Biology	3.2 ± 0.4	0.32 ± 0.1	ng/ml	10.2
2006	Clinical and Experimental Pharmacology and Physiology	15.86 ± 1.38	5.12 ± 0.68	μU/mL	9.9
2006	Journal of Health Sciences	16.54 ± 1.07	5.27 ± 0.76	μU/mL	12.2
2006	Molecular and Cellular Biochemistry	13.67 ± 1.04	6.89 ± 0.22	μU/mL	9.1
2006	Basic & Clinical Pharmacology & Toxicology	13.67± 1.04	6.89± 0.22	μU/mL	9.1
2006	Clinical and Experimental Pharmacology and Physiology	16.6 ± 2.1	4.3 ± 1.3	μU/mL	7.1
2006	Diabetes	0.90 ± 0.09	0.58 ± 0.09	ng/ml	3.5
2006	Phytotherapy Research	2.49 ± 0.26	0.44 ± 0.0	ng/ml	14.6
2007	International Journal of Biological Macromolecules	13.88 ± 14.52	4.87 ± 0.53	μU/mL	0.8
2007	Journal of Ethnopharmacology	296.21 ± 50.40	69.89 ± 10.12	pM/L	6.2
2008	Experimental Diabetes Research	11.8 ± 2.93	3.97 ± 0.86	mIU/L	3.64
2008	BMC Molecular Biology	1.6 ± 0.3	0.7 ± 0.3	ng/ml	3
2008	Atherosclerosis	1.82 ± 0.36	0.05 ± 0.03	μg/L	7.3
2009	Clinical and Experimental Ophthalmology	2.23±0.18	0.99±0.31	ng/ml	4.96
2010	Phytomedicine	38.6±3.8	8.2±1.4	μmol/mL	10.6
2010	Pharmacognosy Res.	390.87 ± 1.18	420.25 ± 2.8	mg/dl	13.7 (-)
2010	Archives of Medical Research	0.67±0.10	0.18 ±0.01	ng/ml	7
2011	Chemico-Biological Interactions	16.55 ± 1.17	6.07 ± 0.99	μU/mL	9.7
2012	International Journal of Endocrinology	38 ± 6	16 ± 2	μU/mL	4.9
2012	The Journal of Pharmacology And Experımental Therapeutıcs	1.69 ± 0.09	0.29 ± 0.03	ng/dl	23
2012	*West Virginia University, School of Medicine*	1.92±0.17	0.47±0.06	ng/ml	6.1
2012	Turkish Journal of Medical Sciences	4.28 ± 0.83	0.12 ± 0.02	ng/ml	7.1
2013	BMC Complementary and Alternative Medicine	14.2 ± 0.583	3.6 ± 0.509	μU/mL	19.6
2013	Diabetology & Metabolic Syndrome	4.68± 0.84	0.65 ±0.14	μU/mL	6.7
2014	BMC Pharmacology and Toxicology	0.31 ± 0.05	0.17 ± 0.04	ng/ml	2.8
2014	Acta Histochemica	15.41 ± 1.21	8.37 ± 1.01	μU/mL	6.3
2014	European Journal of Pharmacology	16.25±1.85	5.02±0.43	μU/mL	8.3
2014	Phytomedicine	15.6 ± 0.5	6.3 ± 0.26	μIU/mL	23.8
2014	Pain Medicine	4.26 ± 0.59	2.28 ± 0.32	μU/mL	4.3
2014	Pain Medicine	4.26 ± 0.59	2.20 ± 0.30	μU/mL	4.5
2014	Pain Medicine	4.26 ± 0.59	2.04 ± 0.42	μU/mL	4.4
2014	Food and Chemical Toxicology	15.9 ± 1.3	26.1 ± 1.4	μU/mL	7.5 (-)
2015	International Journal of Experimental Pathology	6.18± 0.01	0.95 ±0.12	ng/ml	65.3
2015	Pharmacognosy Research	17.66± 2.91	83.33± 6.33	μU/mL	13.3 (-)
2015	Nutrition	53.42±3.73	41.64±2.91	μU/mL	3.5
2015	Renal Failure	8.40 ± 0.34	2.50 ± 0.38	ng/ml	16.8

**Appendix Table 3:** Titles of the publications

Publication Date	Titles of the publications
2005	Streptozotocin-induced diabetes in the rat is associated with enhanced tissue hydrogen sulfide biosynthesis
2005	Quercetin, a flavonoid antioxidant, prevents and protects streptozotocin-induced oxidative stress and -cell damage in rat pancreas
2005	Study of the hypoglycaemic activity of Lepidium sativum L. aqueous extract in normal and diabetic rats
2005	Red wine prevents brain oxidative stress and nephropathy in streptozotocin-induced diabetic rats
2006	Beneficial effects of Aloe vera leaf gel extract on lıpıd profıle status in rats wıth streptozotocın diabetes
2006	Anti-diabetic activity of fruits of terminalia chebula on streptozotocin induced diabetic rats
2006	Rutin improves the antioxidant status in streptozotocin-induced diabetic rat tissues
2006	Antihyperglycaemic and antioxidant effect of rutin, a polyphenolic flavonoid, in streptozotocin-induced diabetic wistar rats
2006	Biochemical evaluation of antidiabetogenic properties of some commonly used Indian plants on streptozotocin-induced diabetes in experimental rats
2006	Chronic inhibition of dipeptidyl peptidase-4 with a sitagliptin analog preserves pancreatic -cell mass and function in a rodent model of type 2 diabetes
2006	Effect of Japanese radish (Raphanus sativus) sprout (Kaiware-daikon) on carbohydrate and lipid metabolisms in normal and streptozotocin-induced diabetic rats
2007	Protective effect of Lycium barbarum polysaccharides on streptozotocin-induced oxidative stress in rats
2007	Effect of Sclerocarya birrea (Anacardiaceae) stem bark methylene chloride/methanol extract on streptozotocin-diabetic rats
2008	The Characterization of High-Fat Diet and Multiple Low-Dose Streptozotocin Induced Type 2 Diabetes Rat Model
2008	Genomic actions of 1,25-dihydroxyvitamin D3 on insulin receptor gene expression, insulin receptor number and insulin activity in the kidney, liver and adipose tissue of streptozotocin-induced diabetic rats
2008	Mechanisms underlying recoupling of eNOS by HMG-CoA reductase inhibition in a rat model of streptozotocin-induced diabetes mellitus
2009	Effect of N-acetylcysteine on the early expression of inflammatory markers in the retina and plasma of diabetic rats
2010	Insulin mimetic impact of Catechin isolated from Cassia fistula on the glucose oxidation and molecular mechanisms of glucose uptake on Streptozotocin-induced diabetic Wistar rats
2010	Antihyperglycemic activity of *Catharanthus roseus* leaf powder in streptozotocin-induced diabetic rats
2010	Effect of Dipeptidyl Peptidase-IV (DPP-IV) Inhibitor (Vildagliptin) on Peripheral Nerves in Streptozotocin-induced Diabetic Rats
2011	Insulin-secretagogue, antihyperlipidemic and other protective effects of gallic acid isolated from Terminalia bellerica Roxb. in streptozotocin-induced diabetic rats
2012	Intermittent Fasting Modulation of the Diabetic Syndrome in Streptozotocin-Injected Rats
2012	Dipeptidyl Peptidase IV Inhibitor Attenuates Kidney Injury in Streptozotocin-Induced Diabetic Rats
2012	Examination of novel cardiac mechanisms ınfluencing mitochondrial proteomes during diabetes mellitus
2012	Effects of lycopene on plasma glucose, insulin levels, oxidative stress, and body weights of streptozotocin-induced diabetic rats
2013	Anti-diabetic, anti-oxidant and anti-hyperlipidemic activities of Melastoma malabathricum Linn. leaves in streptozotocin induced diabetic rats
2013	The effect of a novel curcumin derivative on pancreatic islet regeneration in experimental type-1 diabetes in rats (long term study)
2014	CNX-011-67, a novel GPR40 agonist, enhances glucose responsiveness, insulin secretion and islet insulin content in n-STZ rats and in islets from type 2 diabetic patients
2014	β-Caryophyllene, a natural sesquiterpene, modulates carbohydrate metabolism in streptozotocin-induced diabetic rats
2014	Fisetin improves glucose homeostasis through the inhibition of gluconeogenic enzymes in hepatic tissues of streptozotocin induced diabetic rats
2014	Efficacy of natural diosgenin on cardiovascular risk, insulin secretion, and beta cells in streptozotocin (STZ)-induced diabetic rats
2014	Establishment of a Rat Model of Type II Diabetic Neuropathic Pain
2014	Polyphenols-rich Cyamopsis tetragonoloba (L.) Taub. beans show hypoglycemic and β-cells protective effects in type 2 diabetic rats
2015	Effect of strawberry (Fragaria 3 ananassa) leaves extract on diabetic nephropathy in rats
2015	Anti-diabetic effects of ethanol extract of *Bryonia laciniosa* seeds and its saponins rich fraction in neonatally streptozotocin-induced diabetic rats
2015	Vitamin K1 alleviates streptozotocin-induced type 1 diabetes by mitigating free radical stress, as well as inhibiting NF-kB activation and iNOS expression in rat pancreas
2015	The effects of transdermal insulin treatment of streptozotocin-induced diabetic rats on kidney function and renal expression of glucose transporters

### Calculation of Effect Sizes and Analysis

In this study plasma insulin levels measured in control and STZ groups were considered for calculating effect size values. The effect size of differences regarding plasma insulin levels were accepted as an indicator of practical importance of the differences, therefore one Cohen d formula was used to calculate effect sizes [21, 22].

d= M_1_-M_2_ /√[σ_1_^2^_+_ σ_2_^2^/2] for independent measures

After individual effect sizes per difference in each publication were calculated, mean effect size value was obtained by adding all effect sizes and dividing total effect size score into number of individual effect sizes. Hence just only one value regarding effect of STZ on plasma insulin levels was gathered.

## Results

Results of the study showed that only 4 of the all individual effect sizes indicated negative values while the rest of effect sizes (n=35) was positive. Moreover one small and 38 large effect sizes were seen in the calculations. Descriptive values regarding Plasma Insulin Levels in control and STZ-induced diabetes groups, Unit of Plasma Insulin Levels and Individual Effect Sizes were shown in Table 2.

As seen in the Table 2, the individual effect sizes were between -13.7 to +65.3. The mean effect size value was found as +9.33.

## Discussion

The results of this study made it clearer that STZ-application is an effective way of decreasing significantly plasma insulin levels of rats and mice. Mean effect size value calculated from the publications showed that practical importance of STZ-induced decrease in plasma insulin levels had a large effect. In other words effect size value of +9.33 refers to a large effect size [23]. Therefore the mean value of the STZ applied group is over 90 percentile of the no treatment group or control group.

The results of the study are in line with the findings of the current research studies using STZ for inducing diabetes in rats and mice [9, 10]. Sai Varsha, Thiagarajan, Manikandan and Dhanasekaran applied STZ (35mg/kg) to Male albino Wistar rats, the authors observed plasma insulin decrease in rats after 72 hours [24].

The findings of this study contribute to our understandings about practical importance of differences in plasma insulin levels induced by STZ. When looked at the number of the publications in this study, it can be seen that decisions are based on differences in the publications over 35. Hence the findings of this study make our inferences about plasma insulin level differences induced by STZ more valid rather than relying on only one study’s finding. At the same time findings of the study has a potential for informing researchers about dose and duration of STZ application to change plasma insulin levels of diabetic rats and mice. As another implication of this study, the publications analyzed in this study show characteristics of current practice about using STZ, therefore the effect sizes reported in this study also inform practice using STZ in diabetes studies.

In spite of strong sides of this study, it can be suggested that number of the publications using STZ might be increased in future studies to improving quality of inferences and to make the analysis more comprehensive. At the same time, other publications involving reports and unpublished documents should also be investigated for determining effect sizes regarding the differences about plasma insulin levels induced by STZ. Finally future studies might look at the studies published before 2005.
